# WE3DS: An RGB-D Image Dataset for Semantic Segmentation in Agriculture

**DOI:** 10.3390/s23052713

**Published:** 2023-03-01

**Authors:** Florian Kitzler, Norbert Barta, Reinhard W. Neugschwandtner, Andreas Gronauer, Viktoria Motsch

**Affiliations:** 1 Department of Sustainable Agricultural Systems, Institute of Agricultural Engineering, University of Natural Resources and Life Sciences Vienna, Peter-Jordan-Straße 82, 1190 Vienna, Austria; 2 Department of Crop Sciences, Institute of Agronomy, University of Natural Resources and Life Sciences Vienna, Konrad Lorenz-Straße 24, 3430 Tulln an der Donau, Austria

**Keywords:** crop farming, weed detection, semantic segmentation, image dataset, RGB-D, stereo vision

## Abstract

Smart farming (SF) applications rely on robust and accurate computer vision systems. An important computer vision task in agriculture is semantic segmentation, which aims to classify each pixel of an image and can be used for selective weed removal. State-of-the-art implementations use convolutional neural networks (CNN) that are trained on large image datasets. In agriculture, publicly available RGB image datasets are scarce and often lack detailed ground-truth information. In contrast to agriculture, other research areas feature RGB-D datasets that combine color (RGB) with additional distance (D) information. Such results show that including distance as an additional modality can improve model performance further. Therefore, we introduce WE3DS as the first RGB-D image dataset for multi-class plant species semantic segmentation in crop farming. It contains 2568 RGB-D images (color image and distance map) and corresponding hand-annotated ground-truth masks. Images were taken under natural light conditions using an RGB-D sensor consisting of two RGB cameras in a stereo setup. Further, we provide a benchmark for RGB-D semantic segmentation on the WE3DS dataset and compare it with a solely RGB-based model. Our trained models achieve up to 70.7% mean Intersection over Union (mIoU) for discriminating between soil, seven crop species, and ten weed species. Finally, our work confirms the finding that additional distance information improves segmentation quality.

## 1. Introduction

Scene understanding is an important computer vision concept in applying smart farming (SF) technologies, i.e., vegetation segmentation [1], detecting the composition of plant species in crop farming [2], segmentation of different plant parts [3], or anomalie [4] and disease detection [5]. Automated guided vehicles (AGV) or autonomous mobile robots (AMR), also called field robots, can be used to monitor the status of the crops in high temporal and spatial resolutions and perform precise actions on the field. With a ground-based AGV or AMR equipped with image sensors and accurate computer vision systems, it is possible to obtain parameters of single plants as well as soil heterogeneity and use them for intelligent agricultural processes, such as robotic weed regulation [6] or site-specific fertilization [7] and plant protection [8]. One goal of such SF applications is the reduction of input resources, e.g., water, energy, and agrochemicals, while keeping or even increasing yields [7,9]. The major factor for yield loss is uncontrolled weeds [10] that compete with crops for resources such as nutrients, water, and light. The increasing occurrence of herbicide-resistant weeds [11], as well as negative impacts on the ecosystem and human health [12] makes it necessary to reduce herbicide usage. Spot spraying systems [13,14], mechanical target hoeing [15], or tube stamp robots [16] are options to achieve this goal. However, keeping specific weeds could benefit agricultural sites, for example by nitrogen fixation, erosion protection, or increasing biodiversity [17]. Selective removal of weeds must rely on expert knowledge about plant-crop interactions [18] and computer vision systems that are capable of detecting weed species to distinguish between harmful and harmless weeds. This also includes the necessity of precise localization of different weed species, plant cover, and biomass estimation as well as determining the growth stage of the crop. A computer vision task, that is used for simultaneous classification and localization is called semantic segmentation. The goal of semantic segmentation is to assign a class to each pixel of an image. In an agricultural context, the used classes are often soil, plant residues, or different plant species. The output of a semantic segmentation model is a color-coded segmentation mask, where each color represents a different class. The differently colored areas show the diverse plant composition in the image and the mask can be used to localize the plants on the field to perform actions. Most state-of-the-art semantic segmentation applications in agriculture [19,20,21,22] are based on RGB imagery and distinguish between three classes, i.e., soil, crop, and weeds (all non-crop plant species). The used methods range from machine learning (ML) methods such as Random Forest (RF) based on feature extraction [20] to convolutional neural networks (CNN), such as U-net [23] or fully convolutional network (FCN) [21]. With the growing computational power of embedded computers with a graphics processing unit (GPU), field robots can perform real-time field actions based on semantic segmentation models using state-of-the-art CNNs.

Our goal is to perform a multi-class plant species semantic segmentation under natural light conditions. The challenges are caused by the difficult outdoor environment (different lighting conditions, changing soil and plant appearance) and the complex biological system (many plant species, different growth stages, and supply status). Therefore, a large image dataset is needed to train state-of-the-art semantic segmentation models. First implementations have limited the number of classes to soil, crops and weeds [19,20,21,24] or tried to standardize the light conditions by blocking out the sunlight and artificially illuminating the plants [21,22]. Nevertheless, those models are restricted to the given constraints and can not be used for a broader plant composition analysis or selective removal application. As shown in the literature [25,26], additional distance information can help to achieve better results for semantic segmentation of indoor environments and cityscapes. It is plausible to assume, that this result also holds for multi-class plant species semantic segmentation. For that reason and the lack of publicly available image datasets in agriculture, we collected and annotated images from crops and weeds in early growth stages under real outdoor conditions. To capture a 4-channel color and distance image (RGB-D), we used two cameras in a stereo setup. By adding ground-truth segmentation masks for selected RGB-D images, we introduce a novel RGB-D image dataset, called WE3DS. Finally, we provide a benchmark result for multi-class plant species semantic segmentation and compare models based on RGB, RGB-D and D trained on our developed WE3DS RGB-D image dataset.

The article is structured as follows: Section 2 reviews comparable RGB-D image datasets in other domains and related agricultural image datasets. In Section 3, we describe the development of the WE3DS image dataset including RGB-D sensor and data collection and give an overview of the dataset statistics. Section 4 describes the bechnmark design and results of our multi-class plant species semantic segmentation model. A discussion of the significance of our WE3DS image dataset and our semantic segmentation results is given in Section 5. Section 6 concludes the work and gives an outlook on future research topics.

## 2. Related Work

An RGB-D sensor captures RGB-D images that combine color information (RGB) with depth information (D) to create a detailed 3D representation of an object or scene. We use the term distance to indicate, that the intensity value of the depth map is encoded to represent the distance from an object in the scene to the camera. Another depth map representation encodes the disparity, i.e., pixel parallax of an object between the left and right camera image, of a stereo camera system. Our research focus is on outdoor applications. However, it is worth noting that early implementations of RGB-D image datasets primarily focused on indoor environments and utilized active sensors, such as the Microsoft Kinect, that emit structured light patterns to measure distance. Examples of such datasets are SUNRGB-D [27] and NYUv2 [28]. For outdoor environments, structured light methods are less stable because of exterior lighting conditions [29]. In these environments, passive settings such as stereo sensors are preferable. Cordts et al. published an RGB-D image dataset CityScapes [30] of inner city street scenes captured with a stereo camera setup mounted on top of a driving car. They used two RGB cameras with 2 MP CMOS sensors (OnSemi AR0331) to capture RGB-D images in 50 cities. The final dataset contains 5000 densely labeled and 20,000 coarsely labeled RGB-D images with 19 classes. Other similar RGB-D datasets of street scenes are KITTI Vision Benchmark Suite [31] (400 images, 35 classes), CamVid [32] (701 images, 32 classes), or Daimler Urban Segmentation [33] (500 images, 5 classes).

Image datasets for various computer vision tasks are also available in the field of agriculture. They are collected manually [19,24], with robotic devices [34,35], or by unmanned aerial vehicles (UAV) [20]. Wu et al. [36] have collected weed detection methods based on computer vision and summarized the advantages and disadvantages of various methods and give an overview of available datasets.

An agricultural image dataset collected with the field robot BoniRob [37], which is meant to tackle several tasks including plant classification, localization and mapping was published by Chebrolu et al. [35]. The authors collected a large-scale agricultural robot dataset on sugar beet fields in 2016 using a 4-channel multi-spectral, color (RGB) and near infrared (NIR), camera in an opaque shroud, a Kinect RGB-D sensor under natural light conditions, multiple lidars and Global Navigation Satellite System (GNSS) sensors as well as wheel encoders. They provide ground-truth annotation for 300 RGB-NIR images taken with a JAI AD-130GE camera with a total of 10 classes (sugar beet and 9 different weed species). The dataset was used by Milioto et al. [22] to perform a crop/weed semantic segmentation based on the RGB-NIR images and pre-calculated feature maps.

The Ladybird Cobbitty 2017 Brassica Dataset [34] is a collection of data acquired with the Ladybird field robot, which is equipped with stereo color, thermal and hyperspectral imagery, combined with environmental data collected by a weather station and soil sensor networks and manually added ground-truth data regarding crop status. The data was captured at field trials covering cauliflower and broccoli in different management systems regarding fertilization and irrigation. Bender et al. [38] also provide benchmark results for the task of semantic segmentation (discriminating between broccoli, weeds and soil). They used a multi-class, sparse variational Gaussian process classifier [39] to perform semantic segmentation on pixel-level based on hyperspectral information (400–1000 nm). Therefore, their ground-truth annotation was limited to selected pixels of the plant and no densely annotated data is provided. Furthermore, the RGB-D images were not used for the semantic segmentation task. The distance map was also not used in the benchmark task for object detection. Here they used bounding box annotations of 1248 images of the left camera together and the Faster R-CNN model [40] to detect cauliflower, broccoli and five calibration panels. The distance map is not provided, but the image dataset contains both the left and right camera images with additional intrinsic and extrinsic camera calibration results.

The availability of sufficiently large RGB-D image datasets with dense multi-class semantic annotation has recently enhanced RGB-D semantic segmentation implementations [25,26,41,42,43,44,45] and lead to improvements in model architectures regarding effectiveness and segmentation quality for indoor and outdoor scene understanding. By introducing our WE3DS RGB-D image dataset, we expect a similar improvement in semantic segmentation quality for crop farming, where the biggest challenge is to distinguish similar plants in early growing stages. Additionally, the depth information can be used for further plant status analysis, such as biomass approximation or determining grasp positions for automated actions on the field. By now, no large-scale RGB-D image dataset in the field of crop farming has been published that covers various crop and weed species in early growth stages captured under natural lighting conditions.

## 3. WE3DS Dataset

### 3.1. RGB-D Sensor

**Figure 1:**
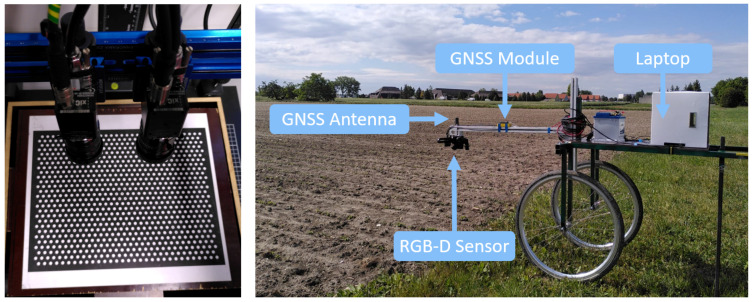
RGB-D sensor with calibration target (**left**) and measurements trolley equipped with sensors on the field (**right**).

For high flexibility, we chose to use two industrial RGB cameras (XIMEA MC023CG-SY) in a stereo camera setup. Each camera contains a 2.3 MP Sony IMX174 LLJ-C sensor with global shutter and a general-purpose input/output (GPIO) connector. We used the XIMEA Application Programming Interface (xiAPI) to trigger the right camera with a software trigger and set up the left camera to be triggered using a synchronization cable and the GPIO connector. The cameras were screwed on a mounting bracket with a baseline of 4 to 5 cm (see Figure 1 left). We used manual iris and focus controlled lenses at 12 mm focal length (TAMRON M112FM12). The equipment reached a spatial ground resolution of 0.4 mm pixel${}^{-1}$ and a ground depth accuracy of 1.6 mm when using it at a working height of 90 cm. Table S2 provides detailed information on the depth accuracy of our RGB-D sensor that depends on the baseline and working distance.

### 3.2. Acquisition Setup

The RGB-D sensor was mounted, top-down heading, on a two-wheeled measurements trolley, which served as a carrier vehicle for hardware and sensors (see Figure 1 right). The wheel distance was fixed at 60 cm to cover common row spacing of different crops. It was equipped with a 12 V battery for power supply, a laptop running our in-house control software, and an Emlid Reach M2 module as GNSS with an antenna mounted above the RGB-D sensor. A stereo camera calibration was performed indoors for each measurement date, to obtain both the intrinsic and extrinsic camera system parameters using the C++ interface of the image analysis tool HALCON. In the calibration process the RGB-D sensor was used to capture ten image pairs (left and right camera image) of a calibration target (see Figure 1 left) in different poses, i.e., position and orientation, and our in-house software calculated camera distortions and relative pose of the cameras. Details about the stereo calibration algorithms that are used by HALCON can be found in [46]. All images were captured with a fixed F-number and pre-set exposure time. At the parcel site, the cameras were focused and white balance was adjusted to the natural light conditions. The exposure time was set using the graphical user interface of the in-house control software and was adjusted if changing light conditions made it necessary. During data collection, the parcel information (parcel ID, plant species, parcel row number) was passed to the in-house software and the acquisition started. While moving the measurements trolley over the crop rows, the cameras captured images at a frame rate of one frame per second (FPS). Depending on the number of parcels ready for collection, the data collection on the field trial took about one hour.

### 3.3. Field Trials

Consecutive field trials were performed in the years 2020 and 2021 on the experimental farm of the University of Natural Resources and Life Sciences, Vienna (BOKU) in Groß-Enzersdorf (48°20’ N, 16°56’ E, 154 m above sea level). The soil is silty loam chernozem of alluvial origin which is rich in calcareous sediments. Each repetition contained small parcels (2.5 m × 9 m in 2020, 1.5 m × 5 m in 2021). After the seedbed preparation, sowing was performed by hand at a depth of 1–2 cm at a row spacing of 50 cm and manual irrigation was performed as required to ensure fast and homogeneous emergence. Thinning of plants and removal of undesired weeds was carried out manually. A parcel contained a single crop species, a single weed species, or a combination of crop and weed species. During seven repetitions (four in 2020, three in 2021) 247 parcels were seeded with 39 different plant species (see Table S1) at three sites of the experimental farm. Due to a high amount of undesired weeds at one site, these parcels could not be used for data acquisition. Other exclusion reasons were too slow or no emergence or damage caused by birds.

### 3.4. Data Collection

The collection of the images started right after the emergence of the plants and until the plants reached a height of 30 cm. Therefore, the specific dates and amount of measurement dates differed for each parcel. Data acquisition was started separately for each parcel. In addition to the stereo image pair (right and left RGB image), a system timestamp, the geolocation (longitude, latitude, UTC), and all camera parameters read by the xiAPI, such as exposure time, image resolution, image format, and white balance factors were recorded. Manually collected metadata (camera mounting height, used cameras, light conditions, wind) was also collected for each measurement date. In total, we collected 6224 image pairs on 25 measurement dates from 84 different parcels.

### 3.5. Data Pre-Processing

**Figure 2:**
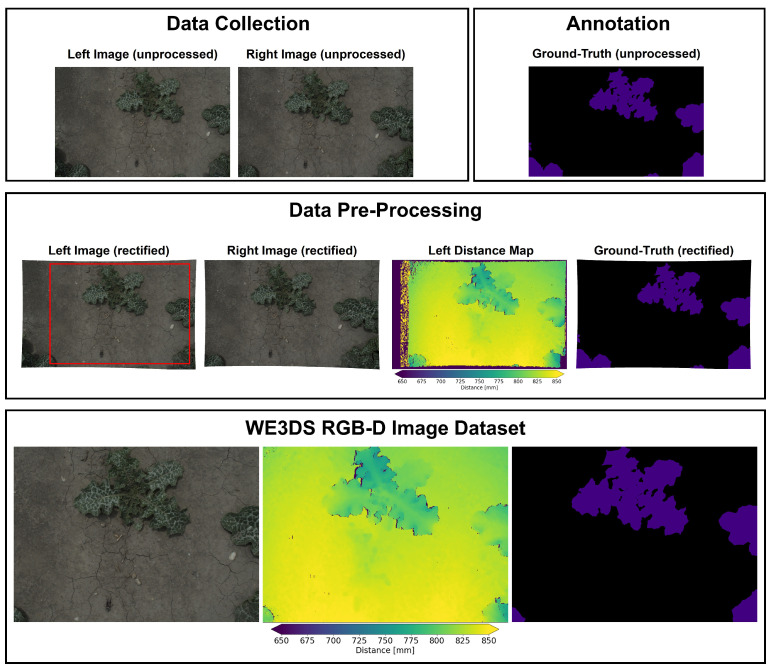
Overview of workflow including data collection (**top**, **left**), ground-truth annotation (**top**, **right**), data pre-processing (**middle**) and a final data point within the WE3DS RGB-D image dataset. The red rectangle in the left rectified image indicates the region of interest for the RGB-D image dataset.

We manually selected RGB images of the left camera for the ground-truth annotation, thus excluding images of poor quality and plant absence. The calculation of the distance map is based on the camera calibration, which consists of intrinsic parameters, i.e., focal length, pixel size, and image origin, and extrinsic parameters, i.e., relative position and orientation of the cameras. This is done in three steps: First, the images are rectified, making use of the calibration data to transform the image to an image space of the calibrated stereo setup. In the calibrated stereo setup, factors such as lens distortions are corrected and features depicted in the left and right images are located on horizontal epipolar lines. Second, stereo matching is performed on the rectified images by finding corresponding features in the left and right images. Based on the simple structure of the epipolar lines, the search space of correspondence is limited to horizontal lines. We used the method normalized cross-correlation (NCC) as a similarity measure at a mask size of 21 pixels for template matching. The NCC method, although computationally more expensive than the absolute gray value differences (SAD) or the sum of squared gray value differences (SSD), provides better robustness to illumination changes that can appear in outdoor environments. The output of the stereo matching is called disparity, which is the pixel parallax of one feature for the left and right rectified image. This disparity is used in the final step together with the baseline of the calibrated stereo setup, the focal length and the image origins to calculate the distance of a feature to the camera. For the data pre-processing steps, we used the HALCON operators map_image for rectification and binocular_distance for distance map calculation, see [46] for more details. Distance calculation fails for pixels that are not present in both images due to occlusion and where the surface provides insufficient texture for matching. As our top-down mounted stereo camera pair captures the same scene from different locations, the left border region in the left image is missing in the right image and vice versa. Based on the geometry of the stereo setup, the rectified left image and the distance map of the left image were cropped to exclude non-overlapping parts of the scene, resulting in image resolutions of 1600 × 1144 pixels. This image pair (color image and distance map) is finally used for the RGB-D image dataset. Figure 2 illustrates the whole dataset development workflow.

### 3.6. Ground-Truth Annotation

Annotation was performed using the computer vision annotation tool CVAT [47]. A class label for each seeded plant species was provided and the annotators drew labeled polygons around each plant instance of the unprocessed left RGB image. An additional label was used for uncertain cases and unknown plant species. The latter represent very small weeds that were overlooked during the manual weed control. Unknown weeds, as well as plant species with very little annotation data were summarized into a void class that is ignored during subsequent steps. Finally, we rectified the ground-truth annotation segmentation masks and cropped them to the same size as the RGB-D image pair. The final dataset export consisted of 2568 densely annotated semantic label maps containing 17 plant species classes with a sufficient amount of ground-truth annotation and the soil class that represents the background (non-annotated part of the image).

### 3.7. Dataset Statistics

#### 3.7.1. Metadata

All images were captured with a fixed F-number and pre-set exposure time. For quality adjustments on the field, mean intensity of the grayscale equivalent image was computed and displayed on the graphical user interface (GUI). Nevertheless, during the acquisition, changes in the weather conditions occurred (clouds covering the sun, shadow from measurements trolley) that result in overexposed or underexposed images. Thus, the RGB images are very heterogeneous in terms of natural light conditions. We performed image acquisition on 25 measurement dates with four cloudy, twelve sunny and nine days of mixed weather conditions. Mixed weather conditions indicate, that fast changes in illumination conditions happened. We also noted the subjective wind intensity into one of the categories light (ten dates), medium (ten dates) and strong (five dates) in order to identify possible sources of error in the stereo reconstruction. Strong wind could decrease the image quality due to blurring of fastly moving plant leaves, as well as complicate the stereo reconstruction when small time delays between the right and left image capture are given.

#### 3.7.2. Distance Maps

**Figure 3:**
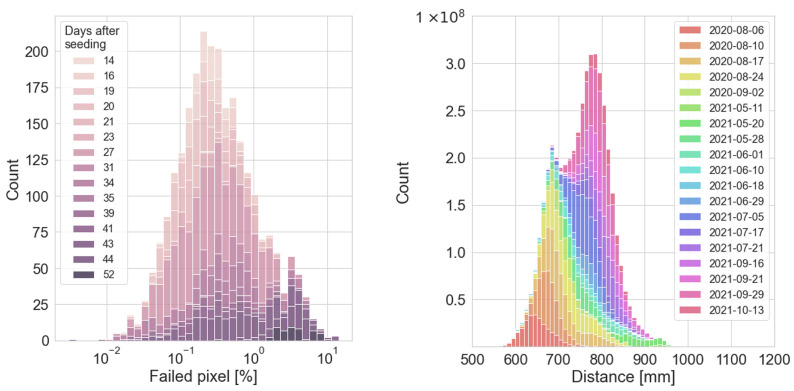
Distribution of failed pixels (in percent) per image colored by days after seeding on logarithmic x-axis (**left**). Distribution of valid distance values (in mm) of the 2568 WE3DS distance maps colored by measurement date (**right**).

Stereo reconstruction is based on the task of finding corresponding features in both images. Due to occlusion and textureless surfaces, there are pixels and regions where distance calculation is not successful. The proportion of those missing distance pixels depends on the scene (small individual plants vs. big occluding plants), image quality and texture of surfaces (different plants have different leaf textures). In Figure 3, (left), the distribution of the proportion of missing pixels is given in percent for the final 2568 RGB-D images and colored by the number of days after seeding. On average there are 0.72% distance pixels missing per image, with a maximum value of 14% for one image. It can be seen that the missing distance pixels are increasing in later stages of measurement and bigger plants. The distance maps encode the distance from the RGB-D sensor to the object in 10${}^{-1}$ mm. Figure 3 (right) shows the distance value distribution of the WE3DS dataset in mm. The color-coding is based on the date of measurement. As the working height of the camera was adjusted according to the plant growth stage, a slight drift of the mean distance can be observed. The dataset has a mean distance of 751.0 mm with a standard deviation of 64.6 mm which was used for distance channel normalization.

#### 3.7.3. Crop and Weeds Distribution

**Figure 4:**
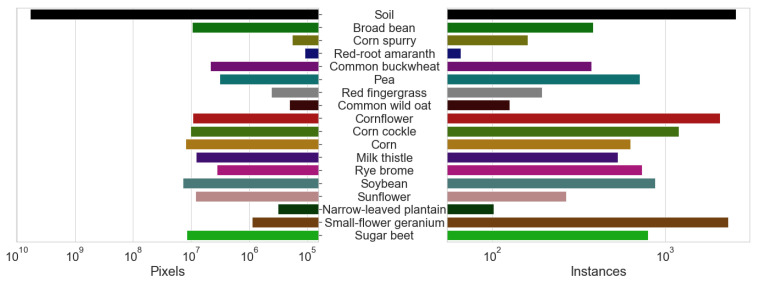
Distribution of labeled pixels (**left**) and instances (**right**) per class for the WE3DS dataset. Both data are shown on a log scale. Instances are the total number of polygons created in the annotation process. No background instances were created during annotation, we assume one instance of background (not annotated area) per image.

Our dataset captured plants in early growth stages. The average plant cover is 2.09% (crops 1.37%, weeds 0.72%). In total, 4038 crop and 7506 weed instances were annotated. On average, an image contains 4.5 individual plants (1.6 crop and 2.9 weed instances) or 38,234 plant pixels (25,131 crop and 13,103 weed pixels). Figure 4 shows the pixel and instance distribution per class on a log scale.

## 4. Benchmark

### 4.1. Task and Metrics

Our benchmark tackles the plant species semantic segmentation task for the WE3DS dataset. In semantic segmentation, the goal is to classify each pixel into a given set of classes, i.e., soil, seven crop species, or ten weed species. The color-coded semantic segmentation map that is the output of such a task, shows the composition and location of different species in an image. We use the Intersection over Union (IoU)

(1) $${\mathrm{IoU}}_{c}=\frac{{\mathrm{TP}}_{c}}{{\mathrm{TP}}_{c}+{\mathrm{FP}}_{c}+{\mathrm{FN}}_{c}}$$

to evaluate per class segmentation quality where ${\mathrm{TP}}_{c}$ is the number of true positives, ${\mathrm{FP}}_{c}$ is the number of false positives, and ${\mathrm{FN}}_{c}$ is the number of false negatives for a class c∈{1,…n}. The mean Intersection over Union (mIoU)

(2) $$\mathrm{mIoU}=\frac{1}{n}\sum_{c=1}^{n}{\mathrm{IoU}}_{c}$$

was used to evaluate the total model performance.

### 4.2. State of the Art

Based on publicly available RGB-D image datasets for indoor environments (SUNRGB-D, NYUv2) and inner city street scenes (CityScapes, KITTI Vision Benchmark Suite), there has been enormous progress in RGB-D semantic segmentation models. First attempts to capture the correlation between color and depth modalities use early concatenation resulting in a 4-channel signal that is used as input [42]. Gupta at al. [48] proposed a new geocentric embedding for depth information encoding the horizontal disparity, height above ground, and angle between surface normal and gravity direction (HHA). Their resulting 3-channel HHA map could be used in model networks that were designed for RGB images [49]. Recently developed models focus on encoder-decoder type models with separate encoder branches for both the RGB image and the distance map and special fusion blocks to combine the color and distance information on different levels of training. Examples of different model architectures for multi-modal feature fusion are RDFNet [45], RedNet [44], ACNet [43], ESANet [26], or the model proposed by Chen et al. [41].

### 4.3. Experiments

The focus of the experiments was to provide a proof of concept for multi-class plant species semantic segmentation using the WE3DS dataset. To achieve this objective, we identified specific criteria for the selection of a model architecture. First, the model should be tested in outdoor environments and achieve state-of-the-art performance compared to the most recently published results in outdoor scenes. Second, we gave preference to models optimized for real-time applicability. Based on these criteria, we selected the ESANet [26] model for our experiments. ESANet was developed for efficient use on mobile platforms and uses ResNet [50] pre-trained on the ImageNet [51] dataset as a backbone architecture for the encoder. The model could achieve state-of-the-art performance for available indoor [27,28] and outdoor [30] datasets and still be able to achieve inference frame rates that can be used for mobile devices. Seichter et al. provided ESANet as a pytorch model and code for model training, evaluation and inference testing for the datasets, see [26].

The WE3DS dataset contains 2568 images and distance maps at a resolution of 1600 × 1140 pixels, which was split into 60% for training and 40% for testing. We added dataset-related code to use ESANet on the WE3DS dataset and performed baseline tests on three different input image resolutions (640 × 480, 1024 × 512, 1280 × 960 pixels). Model training was performed on a GPU (NVIDIA Quadro RTX 8000, 48 GB RAM) for 1500 epochs. The input model resolution of 1280 × 960 pixels, referred to as the full-scale input image, was the highest input resolution we could achieve. Restrictions were given by the network architecture and the RAM of our GPU. The medium-scale (1024 × 512 pixels) and the low-scale (640 × 480 pixels) input resolution models could be trained on a smaller GPU (NVIDIA GeForce RTX 2080, 11 GB RAM). We also provide the memory consumption of the model for training and inference, as well as the inference frame rate in FPS of the trained model. For the latter, inference was performed on 100 test images and the average computation time was calculated. This includes data pre-processing as well as model prediction. Results can be seen in Table 1. The class pixel distribution, see Figure 4, was used for class-specific weights. For comparison, we also included training solely on RGB and D modality.

Information on the dataset and modified code of the ESANet can be found on our website https://doi.org/10.5281/zenodo.7457983 (accessed on 18 December 2022).

### 4.4. Results

**Figure 5:**
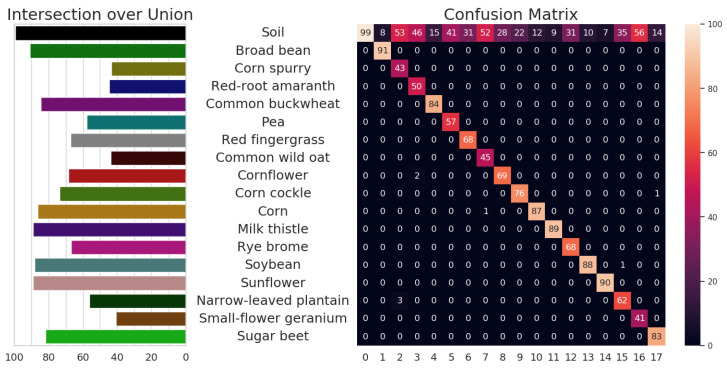
Performance of RGB-D semantic segmentation model trained on full-scale (1280 × 960 pixels) WE3DS dataset. Barplot (**left**) shows per class Intersection over Union IoU${}_{c}$. The confusion matrix (**right**) shows the ground-truth pixels (x-axis) and predicted pixels (y-axis) per class in percent. On the x-axis, 0 stands for class soil and positive integer values refer to the plant IDs, see Table S1.

**Figure 6:**
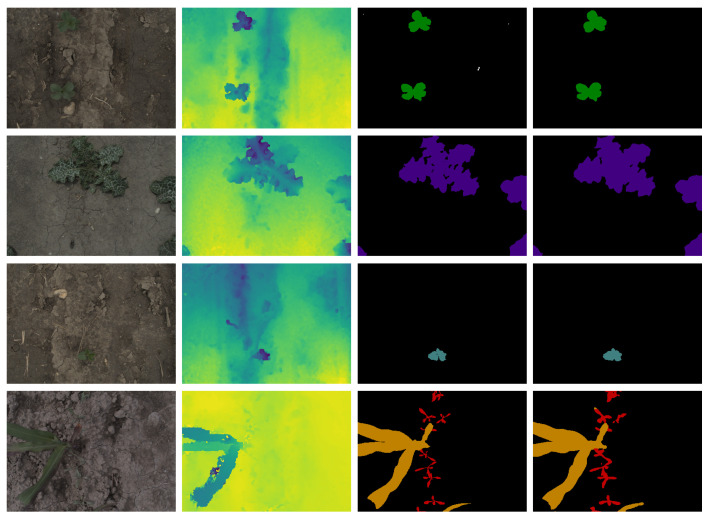
Inference of RGB-D semantic segmentation model trained on full-scale (1280 × 960 pixels) on four exemplary RGB-D images from the test set. Columns (from **left** to **right**) show input RGB image, input distance map with nearest neighbor interpolation, ground-truth annotation and prediction of the trained model. Rows (from **top** to **bottom**) show images taken in broad bean, milk thistle, soybean and combined corn and cornflower parcel. Color codes of the segmentation masks (ground-truth and prediction) correspond to those given in Figure 4, white pixels belong to a void class that was ignored in training and evaluation.

**Figure 7:**
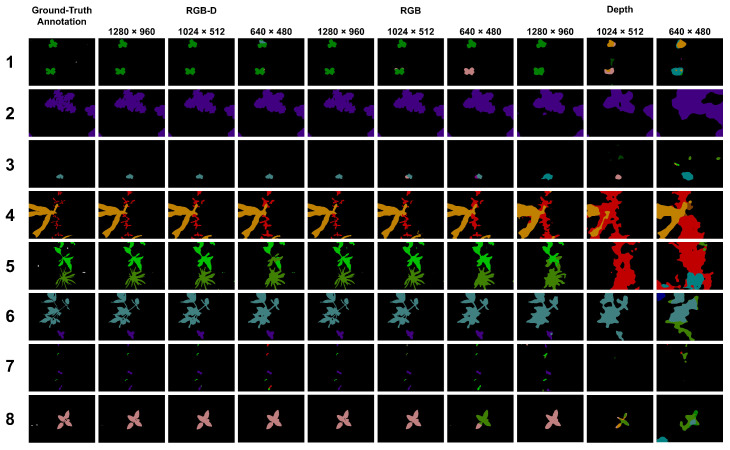
Comparison of semantic segmentation results for different input scales and modalities. From left to right: ground-truth annotation, full-scale (1280 × 960 pixels) RGB-D, medium-scale (1024 × 512 pixels) RGB-D, low-scale RGB-D (640 × 480 pixels), full-scale RGB, medium-scale RGB, low-scale RGB, full-scale depth, medium-scale depth, low-scale depth. Rows (from top to bottom) show images taken in broad bean (1), milk thistle (2), soybean (3), combined corn and cornflower (4), combined sugar beet and corn cockle (5), combined soybean and milk thistle (6), combined sugar beet and milk thistle (7), and sunflower (8). Color codes of the segmentation masks (ground-truth and prediction) correspond to those given in Figure 4, white pixels belong to a void class that was ignored in training and evaluation.

The model parameters and mIoU for the different input image scales and modalities can be found in Table 1. Our best-performing model was the RGB-D ESANet model trained on the full-scale input resolution (1280 × 960 pixels). The model reached the highest mIoU of 70.7% after 1357 epochs and the IoU${}_{c}$ range from 40.7% (small-flower geranium) to 90.9% (broad bean) for the 17 plant species (see Figure 5 left). The soil class is also given with an IoU of 99.5%. The confusion matrix (see Figure 5 right) shows the ground-truth for each class on the x-axis and the prediction of each class on the y-axis. The diagonal shows the precision, also known as the positive predictive value, which is the number of correctly segmented pixels (TP${}_{c}$) in relation to totally segmented class labels of the same class (TP${}_{c}$ + FP${}_{c}$) in percent. It can be seen, that the majority of incorrectly segmented pixels fall into the soil class, which means that the models tend to over-segment the plant parts of the image. This is also reflected in the segmentation output of the models (see Figure 6 and Figure 7). This does not mean, that no plant instance was incorrectly classified, but that the proportion of misclassified plant pixels is small. Tables S3–S11 show that confusion between plant species occurs more often for models with a lower mIoU.

The results in Table 1 also show that models trained solely on distance maps are not performing well for the task of multi-class plant species semantic segmentation. By adding the distance information to the RGB images, the models improved by 4.6% (low-scale), 6.7% (medium-scale), and 0.7% (full-scale) compared to the RGB-only models. Nevertheless, the memory consumption, training time, and inference time increase with adding another modality.

## 5. Discussion

We focused the development of our dataset on the following criteria: (i) collect real crop farming data and realistic plant composition scenes; (ii) focus the image acquisition on early growth stages; (iii) capture images under heterogeneous weather and natural light conditions. The field trials were designed to achieve these goals and the RGB-D sensor was developed for high-throughput data acquisition on the field. The WE3DS dataset consists of 2568 densely annotated RGB-D images of high resolution (1600 × 1140 pixels) and shows real crop farming scenes under natural light conditions. It is the first dataset of this kind and serves as a starting point for the development of a large-scale RGB-D image dataset in agriculture. The images were all captured at the site of the experimental farm in Groß-Enzersdorf which makes the data biased in terms of location. Due to the seven repetitions and different weather conditions during growth and image acquisition, the collected data is heterogeneous in terms of phenological development stages and natural light conditions. Nevertheless, more data from a wider spectrum of plants and locations would be beneficial for providing a more generally usable dataset. The authors see the publication of the WE3DS dataset as a first step to provide widely used RGB-D image datasets in the field of agriculture and crop farming.

Our experiments with different input resolutions simulated the usage of more cost-effective lower resolution RGB-D sensors, we performed the multi-class plant species semantic segmentation model on different input scales. We chose to use 640 × 480 pixels and 1280 × 960 pixels to be able to compare our results to those regarding the SUNRGB-D [27], NYUv2 [28] and CityScapes [30] datasets. Additionally, we used a 1280 × 960 pixels input resolution model as full-scale comparison model. The results in Table 1 show that the full-scale model performs the best in terms of mean Intersection over Union (mIoU) for each modality. This is not surprising, as scaling the image leads to a loss of information mainly for smaller plants (see Figure 7). The full-scale RGB-D model reaches an mIoU of 70.7% for a total of 18 classes (soil, seven crop species, ten weed species). Plant species with a clear signal both in color texture and depth, e.g., broad bean, milk thistle, soybean or sunflower reach an IoU${}_{c}$ of over 85%. The output of the models trained on smaller scale input images do not depict fine leaves and detailed plant structures and tend to over-segment the plant parts, which can also be seen in the confusion matrix, see Tables S3–S11. The distance map alone does not provide enough information to perform multi-class plant species semantic segmentation. Models trained solely on the distance map perform 21.6%(full-scale)–31.8%(medium-scale) worse compared to RGB-based models (see Table 1). Nevertheless, the full-scale model trained on the distance map shows good results for plant species with strong characteristics in the distance map, e.g., broad bean, common buckwheat, milk thistle, and corn, with mIoU${}_{c}$ of up to 77%. On the other hand, plant species with no remarkable characteristics in the distance map are more often confused with other plant species and smaller plants can not be differentiated from the soil (see Figure 7 right). Another observation is that 3D reconstruction works differently for different plant species based on different leaf textures. As can be seen in Figure 6, the distance maps of the milk thistle (second from top) look smooth and precise, whereas the distance map of the corn plant (bottom) shows unsmooth regions and artifacts. One possible explanation is that corn leaves have repetitive patterns of fine parallel leaf veins, making feature matching difficult. Despite the errors in the distance calculation of corn leaves, the distance-based model can recognize the corn plants well. This could be due to the fact that artifacts and erroneous distance values are learned by the model.

The cross-domain comparison, see Table 2, of our semantic segmentation model with those in the literature has limited informative value, as the scenes and number of classes vary. State-of-the-art models reach an mIoU of 51.6% [26] on the NYUv2 and 49.4% [41] on the SUNRGB-D dataset. Both datasets contain low resolution RGB-D images and the models are trained at an input shape of 640 × 480 pixels. When downscaling the WE3DS RGB-D images to such a resolution, we could achieve a comparable mIoU of 48.6%. For the CityScape dataset, an mIoU of up to 75.7% could be achieved by the ESANet [26] on an input resolution of 1024 × 512, whereas our results with the same input resolution could only reach 59.1%. In agriculture, most work has been performed for crop and weeds seperation, leading to a semantic segmentation task with 3 classes (soil, crop, weeds). Milioto et al. [22] could achieve an mIoU of 80.8% (99.5% for soil, 59.2% for weeds, 83.7% for crops) when training and evaluating their model on the BoniRob dataset collected in Bonn [35]. Their proposed model works with a 14-channel input image by pre-computed feature maps and an input resolution of 1296 × 966 pixels. Bosilj et al. [19] could achieve precision values of 99.9% for soil, 66.1% for weeds, and 94.7% for crops on the dataset used in [22] and 98.2% for soil, 80.6% for weeds, and 76.0% for crops on a dataset depicting carrots [24] based on a fully connected CNN model based on color (RGB) and near infrared (NIR) images at an input resolution of 1296 × 966 pixels for [22] and 2428 × 1985 pixels for [24]. Our results provide a first comparative value for multi-class plant species semantic segmentation.

In our benchmark suite, the aim is to develop more accurate and robust multi-class semantic segmentation models for crop farming. It could be shown, that adding the distance map increases the mIoU of the models and that the effect tends to be bigger in small and medium-scale input resolutions. This suggests that it has a greater benefit with cheaper sensors. Furthermore, distance information can be beneficial in smart farming applications not only by increasing the semantic segmentation performance but also by using it for biomass estimation or to generate real-world application points to perform precise actions on the plants or plant parts. This can enable new forms of applications, such as selective removal or multiple cropping systems when combining it with expert knowledge and field robots.

## 6. Conclusions

In this work, we introduced a novel RGB-D image dataset WE3DS, that fills the gap for performing multi-class plant species semantic segmentation. It offers a total of 2568 densely annotated high resolution RGB-D images with 17 plant species. It is the first such RGB-D image dataset to cover real crop farming scenes under natural light conditions. Additionally, 3656 RGB-D images were captured and are planned to be annotated in future work to keep the WE3DS dataset growing. Our work also includes a benchmark suite for evaluation of multi-class plant species semantic segmentation. To compare the characteristics of our WE3DS dataset to similar datasets in other domains, we provide the detailed characteristics of our dataset. Finally, we have performed multi-class plant species semantic segmentation with a state-of-the-art model that achieved up to 70.7% mIoU on the WE3DS dataset, when training it on full-scale RGB-D images. Additionally, we were able to confirm the observation that adding depth information is beneficial in semantic segmentation based on our WE3DS dataset of crop farming scenes. WE3DS presents a new opportunity for scene understanding tasks in the field of crop farming and encourages the improvement of methods for RGB-D semantic segmentation of outdoor scenes under natural light conditions.

Future work includes the expansion of the dataset in terms of additional annotation and including other plant species, as well as adding other kinds of annotations both for 2D and 3D scene understanding tasks, e.g., instance segmentation, keypoints, and 3D annotation. Adding additional RGB-D images from other devices and locations would be beneficial as well to improve the generalization of the model. Additionally, further research could focus on investigating the performance of different model architectures on the WE3DS dataset and on the development of models specifically optimized for agricultural scenes. Such investigations would enhance the current understanding of the challenges regarding plant species semantic segmentation in agricultural environments and could help to identify better-performing models that can be used for practical applications in this domain.

## Figures and Tables

**Table 1:** Performance of multi-class plant species semantic segmentation based on distance (D), color (RGB) and combination of color and distance (RGB-D) for three input image resolutions using ESANet. Model performance parameter is the mean Intersection over Union (mIoU) given in percent. Other model parameters, i.e., inference frame rate in frames per second (FPS), training time in hours, memory consumption during training and inference in GB, were evaluated on an NVIDIA GeForce Quadro RTX 8000. ${}^{†}$ Models were trained on NVIDIA GeForce RTX 2080.

Modality	Input [pixels]	mIoU [%]	Inference Frame Rate [FPS]	Training Time [h]	Inference Memory [MB]	Training Memory [GB]
D	640 × 480	14.4	38.5	42.2 ${}^{†}$	33.3	6.8
RGB	640 × 480	44.0	43.5	44.0 ${}^{†}$	35.8	6.8
RGB-D	640 × 480	48.6	26.5	63.7 ${}^{†}$	51.8	10.8
D	1024 × 512	20.6	27.0	37.7 ${}^{†}$	34.2	11.5
RGB	1024 × 512	52.4	22.2	39.2 ${}^{†}$	38.4	11.5
RGB-D	1024 × 512	59.1	19.2	85.8	55.3	18.5
D	1280 × 960	48.5	11.3	154.1	37.0	27.1
RGB	1280 × 960	70.1	11.0	156.1	46.8	27.1
RGB-D	1280 × 960	70.7	8.6	240.3	66.6	43.4

**Table 2:** Cross-domain comparison of semantic segmentation results. ${}^{†}$ Models show our results with ESANet on the WE3DS dataset (2568 RGB-D images, 18 classes) and reported performances from the literature on the NYUv2 dataset (1449 RGB-D images, 40 classes), SUNRGB-D dataset (10,335 RGB-D images, 37 classes), CityScapes dataset (5000 RGB-D images, 19 classes), and Bonn dataset (10,036 RGB images, 3 classes). Mean Intersection over Union (mIoU) is given in percent. * Model by [22] is an adapted version of SegNet [52] and was trained on a 14-channel input based on RGB information.

Model	Modality	Dataset	Input [pixels]	mIoU [%]
ESANet [26]	RGB-D	NYUv2	640 × 480	51.6
SA-Gate [41]	RGB-D	SUNRGB-D	640 × 480	49.4
ESANet ${}^{†}$	RGB-D	WE3DS	640 × 480	48.6
ESANet [26]	RGB	CityScapes	1024 × 512	72.9
ESANet [26]	RGB-D	CityScapes	1024 × 512	75.7
ESANet ${}^{†}$	RGB	WE3DS	1024 × 512	52.4
ESANet ${}^{†}$	RGB-D	WE3DS	1024 × 512	59.1
ESANet ${}^{†}$	RGB	WE3DS	1280 × 960	70.1
ESANet ${}^{†}$	RGB-D	WE3DS	1280 × 960	70.7
SegNet * [22]	RGB+	Bonn	1296 × 966	80.8
ESANet [26]	RGB	CityScapes	2048 × 1024	77.6
ESANet [26]	RGB-D	CityScapes	2048 × 1024	78.4

## Data Availability

Not applicable.

## Supplementary Materials

The following supporting information can be downloaded at: https://www.mdpi.com/article/10.3390/s23052713/s1, Table S1: Plant species used in the field trials with their plant ID, english names (US) and encoded identifier used by the European and Mediterranean Plant Protection Organization (EPPO); Table S2: Depth accuracy of RGB-D sensor for different baseline setups and for working distances between 0.5 and 1 m; Table S3: Confusion matrix for ESANet trained on RGB-D with input resolution of 1280 × 960 pixels after 1357/1500 epochs; Table S4: Confusion matrix for ESANet trained on RGB-D with input resolution of 1024 × 512 pixels after 1307/1500 epochs; Table S5: Confusion matrix for ESANet trained on RGB-D with input resolution of 640 × 480 pixels after 1415/1500 epochs; Table S6: Confusion matrix for ESANet trained on RGB with input resolution of 1280 × 960 pixels after 1330/1500 epochs; Table S7: Confusion matrix for ESANet trained on RGB with input resolution of 1024 × 512 pixels after 1352/1500 epochs; Table S8: Confusion matrix for ESANet trained on RGB with input resolution of 640 × 480 pixels after 1195/1500 epochs; Table S9: Confusion matrix for ESANet trained on D with input resolution of 1280 × 960 pixels after 1324/1500 epochs; Table S10: Confusion matrix for ESANet trained on D with input resolution of 1024 × 512 pixels after 1087/1500 epochs; Table S11: Confusion matrix for ESANet trained on D with input resolution of 640 × 480 pixels after 1051/1500 epochs.

## Abbreviations

The following abbreviations are used in this manuscript:

AGV	Automated guided vehicles
AMR	Autonomous mobile robots
CVAT	Computer vision annotation tool
CMOS	Complementary metal-oxide-semiconductor
CNN	Convolutional neural network
D	Distance map
FCN	Fully convolutional network
FN	False negatives
FP	False positives
FPS	Frames per second
GNSS	Global navigation satellite system
GPIO	General-purpose input/output
GPU	Graphics processing unit
GUI	Graphical user interface
HHA	Depth information as horizontal disparity, height, angle
mIoU	Mean Intersection over Union
ML	Machine learning
MP	Megapixel
NCC	Normalized cross-correlation
NIR	Near infrared
RAM	Random-access memory
RGB	RGB color space (red, green, blue)
RGB-D	4-channel image (color, distance)
SAD	Sum of absolute gray value difference
SF	Smart farming
SSD	Sum of squared gray value difference
TN	True negatives
TP	True positives
UAV	Unmanned aerial vehicles
UTC	Coordinated Universal Time
xiAPI	XIMEA Application Programming Interface
