# Supplementary material for: WE3DS: An RGB-D Image Dataset for Semantic Segmentation in Agriculture
Source: Sensors (Basel). 2023 Mar 1;23(5):2713. doi: 10.3390/s23052713 (PMC10007111; doi:10.3390/s23052713)
Supplement: Supplementary file 1 [file sensors-23-02713-s001.zip › sensors-2139557-supplementary.pdf]

Supplementary Materials: WE3DS: An RGB-D Image Dataset for Semantic Segmentation in Agriculture

Florian Kitzler [1], Norbert Barta [1], Reinhard W. Neugschwandtner [2], Andreas Gronauer [1] and Viktoria Motsch [1,*]

Table S1. Plant species used in the field trials with their plant ID, english names (US) and encoded identifier used by the European and Mediterranean Plant Protection Organization (EPPO).

Plant ID	EPPO Code	Name (US)	Binomial Name	Type
1	VICFX	Broad bean	*Vicia faba*	crop
2	SPRAR	Corn spurry	*Spergula arvensis*	weed
3	AMARE	Red-root amaranth	*Amaranthus retroflexus*	weed
4	FAGES	Common buckwheat	*Fagopyrum esculentum*	crop
5	PIBSX	Pea	*Pisum sativum*	crop
6	DIGSA	Red fingergrass	*Digitaria sanguinalis*	weed
7	AVEFA	Common wild oat	*Avena fatua*	weed
8	CENCY	Cornflower	*Centaurea cyanus*	weed
9	AGOGI	Corn cockle	*Agrostemma gitahgo*	weed
10	ZEAMX	Corn	*Zea mays*	crop
11	SLYMA	Milk thistle	*Silybum marianum*	weed
12	BROSE	Rye brome	*Bromus secalinus*	weed
13	GLXMA	Soybean	*Glycine max*	crop
14	HELAN	Sunflower	*Helianthus annuus*	crop
15	PLALA	Narrow-leaved plantain	*Plantago lanceolata*	weed
16	GERPU	Small-flower geranium	*Geranium pusillum*	weed
17	BEAVA	Sugar beet	*Beta vulgaris* subsp. *vulgaris* var. *altissima*	crop
18	LACVI	Acrid lettuce	*Lactuca virosa*	weed
19	POAAN	Annual meadowgrass	*Poa annua*	weed
20	BARVU	Bittercress	*Barbarea vulgaris*	weed
21	CAPBP	Blind weed	*Capsella bursa-pastoris*	weed
22	STEME	Chickweed	*Stellaria media*	weed
23	GAETE	Common hemp-nettle	*Galeopsis tetrahit*	weed
24	PAPRH	Common poppy	*Papaver rhoeas*	weed
25	RANRE	Creeping buttercup	*Ranunculus repens*	weed
26	CIRAR	Creeping thistle	*Cirsium arvense*	weed
27	THLAR	Field pennycress	*Thlaspi arvense*	weed
28	FUMOF	Fumitory	*Fumaria officinalis*	weed
29	SETVI	Green foxtail	*Setaria viridis*	weed
30	SSYOF	Hedge mustard	*Sisymbrium officinale*	weed
31	VERHE	Ivy-leaved speedwell	*Veronica hederifolia*	weed
32	POLPE	Ladysthumb	*Polygonum persicaria*	weed
33	HORMU	Mouse barley	*Hordeum murinum*	weed
34	MATMT	Pinapple weed	*Matricaria discoidea*	weed
35	LAMPU	Purple archangel	*Lamium purpureum*	weed
36	VLPMY	Ratstail fescue	*Vulpia myuros*	weed
37	BROMO	Soft brome	*Bromus hordeaceus*	weed
38	CHEAL	White goosefoot	*Chenopodium album*	weed
39	VIOTR	Wild pansy	*Viola tricolor*	weed

Table S2. Depth accuracy of RGB-D sensor for different baseline setups and for working distances between 0.5 and 1 m. The theoretical depth accuracy follows a quadratic function of the distance, with parameters determined by the baseline ($b = 41$ mm or $b = 51$ mm), expected disparity error (0.2 pixel), pixel size (5.86×10^{-3} mm), and focal length (12 mm).

Distance [mm]	Depth Accuracy [mm]	
	$b = 41$ mm	$b = 51$ mm
500	0.6	0.5
550	0.7	0.6
600	0.9	0.7
650	1.0	0.8
700	1.2	0.9
750	1.3	1.1
800	1.5	1.2
850	1.7	1.4
900	1.9	1.6
950	2.1	1.7
1000	2.4	1.9

Table S3. Confusion matrix in percent for ESANet trained on RGB-D with input resolution of 1280 × 960 pixels after 1357/1500 epochs.

	0	1	2	3	4	5	6	7	8	9	10	11	12	13	14	15	16	17
0	99	8	53	46	15	41	31	52	28	22	12	9	31	10	7	35	56	14
1	0	91	0	0	0	0	0	0	0	0	0	0	0	0	0	0	0	0
2	0	0	43	0	0	0	0	0	0	0	0	0	0	0	0	0	0	0
3	0	0	0	50	0	0	0	0	0	0	0	0	0	0	0	0	0	0
4	0	0	0	0	84	0	0	0	0	0	0	0	0	0	0	0	0	0
5	0	0	0	0	0	57	0	0	0	0	0	0	0	0	0	0	0	0
6	0	0	0	0	0	0	68	0	0	0	0	0	0	0	0	0	0	0
7	0	0	0	0	0	0	0	45	0	0	0	0	0	0	0	0	0	0
8	0	0	0	2	0	0	0	0	69	0	0	0	0	0	0	0	0	0
9	0	0	0	0	0	0	0	0	0	76	0	0	0	0	0	0	0	1
10	0	0	0	0	0	0	0	1	0	0	87	0	0	0	0	0	0	0
11	0	0	0	0	0	0	0	0	0	0	0	89	0	0	0	0	0	0
12	0	0	0	0	0	0	0	0	0	0	0	0	68	0	0	0	0	0
13	0	0	0	0	0	0	0	0	0	0	0	0	0	88	0	1	0	0
14	0	0	0	0	0	0	0	0	0	0	0	0	0	0	90	0	0	0
15	0	0	3	0	0	0	0	0	0	0	0	0	0	0	0	62	0	0
16	0	0	0	0	0	0	0	0	0	0	0	0	0	0	0	0	41	0
17	0	0	0	0	0	0	0	0	0	0	0	0	0	0	0	0	0	83

Table S4. Confusion matrix in percent for ESANet trained on RGB-D with input resolution of 1024×512 pixels after 1307/1500 epochs.

	0	1	2	3	4	5	6	7	8	9	10	11	12	13	14	15	16	17
0	99	12	49	34	21	48	45	67	36	30	18	13	40	17	11	46	59	23
1	0	86	0	0	0	0	0	0	0	0	0	0	0	0	5	0	0	0
2	0	0	17	0	0	0	0	0	0	0	0	0	0	0	0	0	0	0
3	0	0	0	63	0	0	0	0	0	0	0	0	0	0	0	0	0	0
4	0	0	0	0	78	0	0	0	0	0	0	0	0	0	0	0	0	0
5	0	0	0	0	0	50	0	0	0	0	0	0	0	0	0	0	0	0
6	0	0	0	0	0	0	54	0	0	0	0	0	0	0	0	0	0	0
7	0	0	0	0	0	0	0	31	0	0	0	0	0	0	0	3	0	0
8	0	0	0	1	0	0	0	0	56	0	0	0	1	0	0	0	0	0
9	0	0	0	0	0	0	0	0	3	67	0	0	2	0	0	12	0	3
10	0	0	0	0	0	0	0	0	0	0	80	0	0	0	0	0	0	0
11	0	0	0	0	0	0	0	0	0	0	0	86	0	0	0	0	0	0
12	0	0	0	0	0	0	0	0	0	0	0	0	55	0	0	0	0	0
13	0	0	0	0	0	0	0	0	0	0	0	0	0	80	1	0	0	0
14	0	0	0	0	0	0	0	0	0	0	0	0	0	1	80	0	0	0
15	0	0	31	0	0	0	0	0	0	0	0	0	0	0	0	36	0	0
16	0	0	0	0	0	0	0	0	0	0	0	0	0	0	0	0	38	0
17	0	0	0	0	0	0	0	0	0	0	0	0	0	0	0	0	0	68

Table S5. Confusion matrix in percent for ESANet trained on RGB-D with input resolution of 640×480 pixels after 1415/1500 epochs.

	0	1	2	3	4	5	6	7	8	9	10	11	12	13	14	15	16	17
0	99	16	45	32	28	53	39	67	45	42	26	17	46	23	17	51	60	26
1	0	81	0	0	0	0	0	0	0	0	0	0	0	3	4	0	0	0
2	0	0	9	0	0	0	0	0	0	0	0	0	0	0	0	0	0	0
3	0	0	0	67	0	0	0	0	0	0	0	0	0	0	0	0	0	0
4	0	0	0	0	69	0	0	0	0	0	0	0	0	0	0	0	0	0
5	0	0	1	0	0	38	0	0	0	0	0	0	0	0	0	0	0	0
6	0	0	6	0	0	0	60	0	0	0	0	0	0	0	0	0	0	0
7	0	0	0	0	0	0	0	22	0	0	0	0	0	0	0	0	0	0
8	0	0	0	0	0	0	0	1	36	0	0	0	5	0	0	4	4	0
9	0	0	15	0	0	4	0	0	11	52	0	0	3	0	1	2	2	2
10	0	0	0	0	0	0	0	2	0	0	69	0	0	0	0	0	0	0
11	0	0	0	0	0	0	0	0	1	0	0	82	0	0	1	0	0	0
12	0	0	0	0	0	0	0	0	0	0	0	0	43	0	0	0	0	0
13	0	0	0	0	0	0	0	0	0	0	0	0	0	68	0	0	0	0
14	0	1	0	0	0	0	0	0	0	1	0	0	0	3	71	0	0	0
15	0	0	20	0	0	0	0	3	0	0	0	0	0	0	0	37	0	0
16	0	0	0	0	0	0	0	1	0	0	0	0	0	0	0	0	28	0
17	0	0	0	0	0	1	0	0	3	2	0	0	0	0	0	1	1	67

Table S6. Confusion matrix in percent for ESANet trained on RGB with input resolution of 1280 × 960 pixels after 1330/1500 epochs.

	0	1	2	3	4	5	6	7	8	9	10	11	12	13	14	15	16	17
0	99	8	51	43	13	40	33	56	27	23	12	9	34	10	8	33	56	13
1	0	91	0	0	0	0	0	0	0	0	0	0	0	0	0	0	0	0
2	0	0	38	0	0	0	0	0	0	0	0	0	0	0	0	0	0	0
3	0	0	0	52	0	0	0	0	0	0	0	0	0	0	0	0	0	0
4	0	0	0	0	86	0	0	0	0	0	0	0	0	0	0	0	0	0
5	0	0	0	0	0	57	0	0	0	0	0	0	0	0	0	0	0	0
6	0	0	0	0	0	0	66	0	0	0	0	0	0	0	0	0	0	0
7	0	0	0	0	0	0	0	40	0	0	0	0	0	0	0	0	0	0
8	0	0	0	4	0	0	0	0	71	0	0	0	0	0	0	0	0	0
9	0	0	0	0	0	0	0	0	0	73	0	0	0	0	0	0	0	0
10	0	0	0	0	0	0	0	1	0	0	87	0	0	0	0	0	0	0
11	0	0	0	0	0	0	0	0	0	0	0	89	0	0	0	0	0	0
12	0	0	0	0	0	0	0	0	0	0	0	0	65	0	0	0	0	0
13	0	0	0	0	0	0	0	0	0	0	0	0	0	88	0	0	0	0
14	0	0	0	0	0	0	0	0	0	0	0	0	0	0	91	0	0	0
15	0	0	10	0	0	0	0	0	0	0	0	0	0	0	0	64	0	0
16	0	0	0	0	0	0	0	0	0	0	0	0	0	0	0	0	42	0
17	0	0	0	0	0	0	0	0	0	0	0	0	0	0	0	1	0	84

Table S7. Confusion matrix in percent for ESANet trained on RGB with input resolution of 1024 × 512 pixels after 1352/1500 epochs.

	0	1	2	3	4	5	6	7	8	9	10	11	12	13	14	15	16	17
0	99	12	49	30	21	49	31	54	37	28	21	15	42	18	13	47	59	21
1	0	84	0	0	0	0	0	0	0	0	0	0	0	0	2	0	0	0
2	0	0	15	0	0	0	0	0	0	0	0	0	0	0	0	0	0	0
3	0	0	0	69	0	0	0	0	0	0	0	0	0	0	0	0	0	0
4	0	0	0	0	78	0	0	0	0	0	0	1	0	0	1	0	0	1
5	0	0	7	0	0	50	0	0	0	0	0	0	0	0	0	0	0	0
6	0	0	0	0	0	0	24	0	0	0	0	0	0	0	0	0	0	0
7	0	0	0	0	0	0	0	38	0	0	0	0	0	0	0	5	0	0
8	0	0	0	0	0	0	19	0	50	0	0	0	11	0	0	3	3	0
9	0	0	1	0	0	0	0	0	8	61	0	0	2	0	0	13	0	2
10	0	0	0	0	0	0	24	6	0	0	76	0	0	0	0	5	0	1
11	0	0	0	0	0	0	0	0	0	0	0	81	0	0	0	0	0	0
12	0	0	0	0	0	0	0	0	0	0	0	0	43	0	0	1	0	0
13	0	0	0	0	0	0	0	0	0	0	0	0	0	79	3	1	0	0
14	0	1	0	0	0	0	0	0	0	3	0	0	0	0	77	0	0	0
15	0	0	24	0	0	0	0	0	0	0	0	0	0	0	0	20	0	0
16	0	0	0	0	0	0	0	0	0	0	0	0	0	0	0	0	33	0
17	0	0	0	0	0	0	0	0	1	1	0	0	0	0	0	1	1	70

Table S8. Confusion matrix in percent for ESANet trained on RGB with input resolution of 640 × 480 pixels after 1195/1500 epochs.

	0	1	2	3	4	5	6	7	8	9	10	11	12	13	14	15	16	17
0	99	15	46	30	28	57	24	70	45	35	28	20	52	23	16	60	66	26
1	0	83	0	0	0	2	0	0	0	7	0	0	0	1	5	0	0	0
2	0	0	14	0	0	0	0	0	0	0	0	0	0	0	0	0	0	0
3	0	0	0	56	0	0	0	0	0	0	0	0	0	0	0	0	0	0
4	0	0	0	0	67	0	0	0	0	0	1	1	0	0	0	0	0	1
5	0	0	4	1	0	37	0	0	0	0	0	0	0	0	0	0	1	0
6	0	0	1	0	0	0	75	0	0	0	0	0	0	0	0	0	0	0
7	0	0	0	0	0	0	0	24	0	0	0	0	0	0	0	0	0	0
8	0	0	1	9	0	0	0	1	32	0	0	0	7	0	0	3	5	1
9	0	0	0	0	0	0	0	0	15	34	0	0	14	0	0	20	1	2
10	0	0	0	0	0	0	0	1	0	0	68	0	0	0	0	0	0	2
11	0	0	0	0	0	0	0	0	1	0	0	73	0	0	0	0	0	0
12	0	0	0	0	0	0	0	0	0	0	0	0	24	0	0	1	0	0
13	0	0	0	0	1	0	0	0	0	4	0	0	0	69	0	0	1	0
14	0	0	0	0	0	0	0	0	0	15	0	1	0	4	77	0	0	0
15	0	0	31	0	0	0	0	1	0	0	0	0	0	0	0	12	0	0
16	0	0	0	0	0	0	0	0	0	0	0	0	0	0	0	0	20	0
17	0	0	0	0	0	0	0	0	2	1	0	0	0	0	0	0	1	63

Table S9. Confusion matrix in percent for ESANet trained on D with input resolution of 1280 × 960 pixels after 1324/1500 epochs.

	0	1	2	3	4	5	6	7	8	9	10	11	12	13	14	15	16	17
0	99	15	54	17	25	54	45	65	44	37	26	18	49	21	16	51	78	22
1	0	81	0	0	1	0	0	0	0	0	0	0	0	0	0	0	0	0
2	0	0	23	0	0	0	0	0	0	0	0	0	0	0	0	3	0	0
3	0	0	0	82	0	0	0	0	0	0	0	0	0	0	0	0	0	0
4	0	0	0	0	71	0	0	0	0	0	0	0	0	0	0	0	0	0
5	0	0	7	0	0	41	0	0	0	0	1	0	0	0	0	0	0	0
6	0	0	0	0	0	0	54	0	0	0	0	0	0	0	0	0	0	0
7	0	0	0	0	0	0	0	32	0	0	0	0	0	0	0	1	0	0
8	0	0	0	0	0	0	0	0	48	7	0	0	2	0	0	15	0	0
9	0	0	0	0	0	0	0	0	1	49	0	0	0	0	0	0	0	2
10	0	0	0	0	0	0	0	0	0	0	70	0	0	0	0	0	0	0
11	0	0	0	0	0	0	0	0	0	0	0	81	0	0	0	0	0	0
12	0	0	0	0	0	0	0	0	0	0	0	0	45	0	0	0	0	0
13	0	1	9	0	0	1	0	0	0	0	0	0	0	75	1	0	0	0
14	0	0	0	0	0	0	0	0	0	0	0	0	0	1	80	0	0	0
15	0	0	3	0	0	0	0	0	0	2	0	0	0	0	0	18	0	0
16	0	0	0	0	0	0	0	0	0	0	0	0	0	0	0	1	19	0
17	0	0	0	0	0	0	0	1	3	3	0	0	0	0	0	7	1	74

Table S10. Confusion matrix in percent for ESANet trained on D with input resolution of 1024 × 512 pixels after 1087/1500 epochs.

	0	1	2	3	4	5	6	7	8	9	10	11	12	13	14	15	16	17
0	99	17	53	0	28	41	51	0	60	51	28	47	79	32	18	84	100	32
1	0	80	2	0	2	33	0	0	0	0	4	0	0	9	9	1	0	0
2	0	0	0	0	0	0	0	0	0	0	0	0	0	0	0	0	0	0
3	0	0	0	0	0	0	0	0	0	0	0	0	0	0	0	0	0	0
4	0	0	8	0	66	4	0	0	0	0	0	0	0	0	18	2	0	0
5	0	0	8	0	0	14	0	0	0	0	2	0	0	0	0	1	0	0
6	0	0	0	0	0	0	42	0	0	0	0	0	0	0	0	0	0	0
7	0	0	0	0	0	0	0	0	0	0	0	0	1	0	0	0	0	0
8	0	0	0	0	0	0	0	0	9	13	0	0	3	0	0	0	0	0
9	0	0	1	0	0	0	0	0	9	26	0	0	3	1	0	1	0	0
10	0	0	1	0	0	0	5	0	8	1	57	0	2	0	0	0	0	0
11	0	2	0	0	0	0	0	0	0	0	0	51	0	0	0	1	0	0
12	0	0	0	0	0	0	0	0	4	2	0	0	5	0	0	0	0	0
13	0	0	13	0	0	0	0	0	0	0	2	0	0	37	7	2	0	0
14	0	0	5	0	0	6	0	0	0	0	2	0	0	4	42	1	0	1
15	0	0	3	0	0	0	0	0	0	0	0	0	0	0	0	0	0	0
16	0	0	0	0	0	0	0	0	0	0	0	0	0	0	0	0	0	0
17	0	0	0	0	0	0	0	0	5	2	0	0	2	12	3	1	0	63

Table S11. Confusion matrix in percent for ESANet trained on D with input resolution of 640 × 480 pixels after 1051/1500 epochs.

	0	1	2	3	4	5	6	7	8	9	10	11	12	13	14	15	16	17
0	99	34	69	98	46	80	84	88	95	77	54	49	95	43	33	83	99	54
1	0	31	0	0	6	4	0	0	0	0	2	0	0	2	12	0	0	0
2	0	0	0	0	0	0	0	0	0	0	0	0	0	0	0	0	0	0
3	0	0	0	0	0	0	0	0	0	0	0	0	0	0	0	0	0	0
4	0	1	0	0	31	3	0	0	0	0	0	0	0	0	0	0	0	1
5	0	0	1	0	0	1	1	0	0	0	3	0	0	0	0	14	0	0
6	0	0	0	0	0	0	7	0	0	0	0	0	0	0	0	0	0	0
7	0	0	0	0	0	0	0	0	0	0	0	0	0	0	0	0	0	0
8	0	2	19	0	0	0	4	0	1	4	0	0	0	0	0	0	0	0
9	0	0	1	0	1	2	1	0	0	6	1	0	0	0	0	0	0	2
10	0	0	0	0	0	0	0	4	0	1	28	0	0	0	0	0	0	0
11	0	8	0	0	0	0	0	0	0	0	0	50	0	0	38	0	0	2
12	0	0	0	0	0	0	0	0	0	0	0	0	1	0	0	0	0	0
13	0	5	0	0	5	5	0	0	0	1	4	0	0	39	0	0	0	0
14	0	12	0	0	7	1	0	0	0	0	2	0	0	6	15	0	0	4
15	0	0	6	0	0	0	0	0	0	0	0	0	0	0	0	0	0	0
16	0	0	0	0	0	0	0	0	0	0	0	0	0	0	0	2	0	0
17	0	2	0	0	0	0	0	2	1	4	0	0	0	5	0	0	0	30
